# In-vivo validation of interpolation-based phase offset correction in cardiovascular magnetic resonance flow quantification: a multi-vendor, multi-center study

**DOI:** 10.1186/s12968-019-0538-3

**Published:** 2019-05-20

**Authors:** Mark B. M. Hofman, Manouk J. A. Rodenburg, Karin Markenroth Bloch, Beat Werner, Jos J. M. Westenberg, Emanuela R. Valsangiacomo Buechel, Robin Nijveldt, Onno A. Spruijt, Philip J. Kilner, Albert C. van Rossum, Peter D. Gatehouse

**Affiliations:** 1 Radiology and Nuclear Medicine, ICaR-VU, VU University Medical Center, PO Box 7057, 1007 MB Amsterdam, the Netherlands; 2 Lund University Bioimaging Center, Lund University, SE-221 85 Lund, Sweden; 3 Philips Healthcare, SE-164 85 Stockholm, Sweden; 4 Department Diagnostic Imaging, University Children’s Hospital, Steinwiesstrasse 75, 8032 Zürich, Switzerland; 5 Radiology, Leiden University Medical Center, Albinusdreef 2, 2333 ZA Leiden, the Netherlands; 6 Division of Cardiology, University Children’s Hospital, Steinwiesstrasse 75, 8032 Zürich, Switzerland; 7 Cardiology, ICaR-VU, VU University Medical Center, PO Box 7057, 1007 MB Amsterdam, the Netherlands; 8 Pulmonology, ICaR-VU, VU University Medical Center, PO Box 7057, 1007 MB Amsterdam, the Netherlands; 9 Cardiovascular Magnetic Resonance Unit, Royal Brompton Hospital, Sydney Street, London, SW3 6NP UK

**Keywords:** Flow quantification, Velocity offset, Cardiac output, Phase contrast velocity mapping, Aorta, Main pulmonary artery, MRI, Background offset

## Abstract

**Background:**

A velocity offset error in phase contrast cardiovascular magnetic resonance (CMR) imaging is a known problem in clinical assessment of flow volumes in vessels around the heart. Earlier studies have shown that this offset error is clinically relevant over different systems, and cannot be removed by protocol optimization. Correction methods using phantom measurements are time consuming, and assume reproducibility of the offsets which is not the case for all systems. An alternative previously published solution is to correct the in-vivo data in post-processing, interpolating the velocity offset from stationary tissue within the field-of-view. This study aims to validate this interpolation-based offset correction in-vivo in a multi-vendor, multi-center setup.

**Methods:**

Data from six 1.5 T CMR systems were evaluated, with two systems from each of the three main vendors. At each system aortic and main pulmonary artery 2D flow studies were acquired during routine clinical or research examinations, with an additional phantom measurement using identical acquisition parameters. To verify the phantom acquisition, a region-of-interest (ROI) at stationary tissue in the thorax wall was placed and compared between in-vivo and phantom measurements. Interpolation-based offset correction was performed on the in-vivo data, after manually excluding regions of spatial wraparound. Correction performance of different spatial orders of interpolation planes was evaluated.

**Results:**

A total of 126 flow measurements in 82 subjects were included. At the thorax wall the agreement between in-vivo and phantom was − 0.2 ± 0.6 cm/s. Twenty-eight studies were excluded because of a difference at the thorax wall exceeding 0.6 cm/s from the phantom scan, leaving 98. Before correction, the offset at the vessel as assessed in the phantom was − 0.4 ± 1.5 cm/s, which resulted in a − 5 ± 16% error in cardiac output. The optimal order of the interpolation correction plane was 1st order, except for one system at which a 2nd order plane was required. Application of the interpolation-based correction revealed a remaining offset velocity of 0.1 ± 0.5 cm/s and 0 ± 5% error in cardiac output.

**Conclusions:**

This study shows that interpolation-based offset correction reduces the offset with comparable efficacy as phantom measurement phase offset correction, without the time penalty imposed by phantom scans.

**Trial registration:**

The study was registered in The Netherlands National Trial Register (NTR) under TC 4865. Registered 19 September 2014. Retrospectively registered.

## Background

Measurement of blood flow is potentially an unrivalled asset of cardiovascular magnetic resonance (CMR), and able to measure the volume flow in large vessels by pixel wise mapping of the velocities through planes transecting the vessels. This should provide the most accurate measurements available of aortic or pulmonary regurgitation, cardiac output, shunt flow and, indirectly, of mitral and tricuspid regurgitation [1, 2]. The technique applied clinically for the flow measurements is 2-dimensional (2D) cine phase contrast velocity quantification, using a flow sensitivity perpendicular to the image plane. However, phase contrast velocity mapping remains under-used, and may have become discredited in the eyes of some CMR users, because even when appropriate methods of acquisition have been used, inaccurate flow measurements can be caused by background phase errors [1, 3].

These background phase errors result in offsets in the velocity values, typically in the range up to 4,9 cm/s [4]. However, when velocity values are integrated over the vessel cross-section and over time, this relatively small velocity offset can accumulate into significant errors in volume flow [4, 5]. In most applications around the body these offsets can be corrected by the velocity offset as obtained in directly surrounding stationary tissue. However, for flow assessment in the large vessels around the heart, no stationary tissue is situated close to the vessels and as the velocity offsets vary spatially over the image, corrections based on distant phase (such as the LPC filter described later) are more error prone.

As flow quantification at the aorta and main pulmonary artery are the two main applications for clinical CMR velocity imaging, this study focused on 2D phase contrast imaging of these vessels. It was shown that the velocity offset needs to be below 0.6 cm/s for reliable clinical CMR imaging of the volume flows, typically to obtain an error in cardiac output below 5% [4]. Earlier studies have shown that offsets found at different CMR systems are often larger than this target value [3, 4, 6]. Thus, correction or reduction of this velocity offset is needed.

The cause of the velocity offset is known. A large source is the concomitant field (i.e. Maxwell) terms of the gradient fields [7]. As these can be estimated by the known gradient fields, most commercial CMR systems perform a software correction for these effects nowadays [7]. A second contribution arises from imperfections in the eddy currents compensation (gradient waveform pre-emphasis) [8], where even very minor errors can cause significant velocity offsets. More recently mechanical resonance effects in the gradient coil resulting in vibrations are described as a third contributing factor [9, 10].

The velocity offsets issue led to an initiative backed by the European Association of Cardiovascular Imaging (EACVI) CMR section of the European Society of Cardiology to determine whether these offsets were a general or more site-specific problem. The first study in this initiative showed that these offsets are generic and apparent on systems of all 3 main CMR vendors: General Electric Healthcare, Siemens Healthineers and Philips Healthcare [4]. A second phantom study investigated whether velocity offsets could be avoided by reducing them below 0.6 cm/s with general protocol optimization. In a multi-vendor setup, it was shown that the offset problem can be reduced by protocol settings but cannot be solved by protocol optimization alone [11].

When these offsets are present in the acquisition and are not easily preventable with general protocol guidelines, they should be corrected in post-processing. A fixed correction per CMR system cannot be used as the offsets are dependent on many protocol parameters such as slice orientation, and thus vary per specific acquisition. The most straightforward approach is repeating the identical acquisition on a static phantom to determine the offset, and subtracting the corresponding apparent phantom velocities from those of the clinical acquisition. This approach was performed earlier in several single center studies [3, 6, 12]. Such a correction method assumes temporal stability of the velocity offset over time. This assumption was tested in a multi-vendor study, in which the offset appeared to be stable for most systems, but not for all [13]. Secondly, such a phantom offset correction is time-consuming in a busy clinical schedule as it requires additional scanner time for every single flow acquisition [6].

Another post- processing approach is the estimation of the offset by using the velocity values of stationary tissue within the field-of-view (FOV); a spatially interpolation-based offset correction. Walker et al. proposed an algorithm to detect stationary tissue on a pixelwise basis within the 2D image using a threshold on the standard deviation of the velocity over the cardiac cycle [8]. Using a mask of stationary tissue, the velocity offset was linearly interpolated over the FOV to give a correction at the location of the vessel of interest. More recently, this method was reconfirmed on a newer CMR system, again in a single center study [14]. However, this method has never been validated in a multi-center study across systems from different vendors. Furthermore, there is some debate in the literature whether linear interpolation is the best method for this correction. Some studies applied higher order spatial fitting over the FOV [15–17], whereas other studies found that this resulted in a lower overall accuracy for the measurement at the aorta and main pulmonary artery [14, 18].

The main objective of this study is to validate the interpolation-based offset correction (exactly as described fully in reference [14] based on reference [8]) of the velocity offset errors in the aorta and pulmonary artery within a multi-center, multi-vendor setting of regular routine unaided clinical use of the cine phase-contrast flow technique. A secondary objective is to assess whether linear or higher order (curved)-spatial interpolation is required. Finally, this study describes the velocity error and subsequent error in cardiac output over different CMR systems and sites.

## Methods

### Study inclusions

For this study, 5 sites were selected with 6 whole body 1.5 T CMR systems on which regular aorta and pulmonary flow acquisitions are performed. Two systems were included for each of the 3 main vendors, Table 1 shows the system characteristics.

**Table 1:** MR system characteristics

Vendor	Type	Software version	System nr
Philips	Ingenia / Achieva	R5 / R.3.2	1/2
Siemens	Avanto	VB17	3/4
GE	Signa HDxt / Discovery MR 450	HD23.0 V01 / DV24.0 R01	5/6

2D phase contrast studies were included from patient studies in which aorta and/or the main pulmonary flow assessment were obtained for either clinical indication or within another research protocol. An inclusion criterion was acquisition in a patient with a sinus rhythm. All data sent to the corelab (Image Analysis section below) were anonymized. To keep balance in the data, we aimed to include 10 studies for each vessel on each CMR system.

### CMR acquisition protocol

Each site applied their local protocol for aorta or pulmonary flow acquisitions. For this study, we applied a minimal set of protocol requirements which are known to generally reduce the velocity offset [4, 11], and are clinical practice in most centers. This was to validate the offset correction in a manner most representative for clinical practice.

The applied technique for flow assessment was 2D phase contrast velocity quantification with a spoiled gradient echo imaging pulse sequence in cine mode with retrospective or retrogated electrocardiographic (ECG) triggering. Imaging was performed with table shift such that the center of the FOV was positioned in the transaxial iso-center plane (i.e. at z = 0 where z = the head-foot direction) of the scanner, as supported by the vendor’s software in routine clinical use. The encoding velocity (V_enc_) was adapted reasonably to the peak velocity V_peak_; meaning (0.8 x V_peak_) < V_enc_ < (2 x V_peak_), such that any velocity aliasing was resolved in post processing. Phase errors due to Maxwell/concomitant gradient terms were corrected within the image reconstruction, as was implemented in all the different included CMR systems [7]. Finally, specific requirements as advised by the vendors were included to limit gradient slew rates (General Electric: flow optimization ‘on’. Philips: default gradient mode. Siemens: normal gradient mode).

In order to apply the interpolation-based offset correction method the following additional protocol requirements were added. No offset correction filters from vendors were applied in the image reconstruction (see Discussion); for Philips systems the default background phase-offset correction (‘LPC filter’) and noise clipping were switched off. Spatial wraparound in the phase encoding direction was limited, with instructions that each center should ensure a remaining ‘air gap’ preventing phase-encode wrapped tissue from overlapping onto other tissue in the image. Both posterior and anterior radiofrequency (RF) receiver coils were enabled in the image FOV. If, for example, only anterior RF surface coils were enabled, the resulting low signal to noise ratio (SNR) posteriorly limited the automatic mask calculation in the correction. Finally for validation purposes we required a FOV set such that stationary tissue in the thorax wall was in view.

### Phantom measurements

In order to act as a reference standard for the velocity offsets, measurements in a stationary phantom were applied. After each patient acquisition, a phantom measurement with exactly the same protocol settings was performed within 24 h. A fluid or gelatin filled stationary phantom was imaged, with a size such that it included both the location of the vessel of interest as well as a part of the thorax wall. The gelatin or water phantom was doped to shorten T1 values resulting in a signal to noise ratio in the imaging protocol above muscular tissue. The phantom acquisition was either triggered on a simulated ECG at the same rate as the patient or otherwise at 60 beats/min. For fluid filled phantoms a 5 min waiting time was included after the phantom positioning, to ensure that no residual fluid motion was present during the measurement. This waiting delay was omitted in case of a gelatin filled phantom. For systems (Philips) which showed earlier a dependency of the velocity offset on the gradient system duty cycle of preceding scans [10, 13], pre-scanning was performed before the phantom flow acquisition to ensure similar gradient coil heating. The pre-scanning consisted of the same MR pulse-sequences as were applied in the patient study in the 5 min preceding the clinical flow scan.

Earlier studies have shown that due to system heating, temporal stability of the velocity offset is perturbed in some systems [10, 13]. This instability had the potential to invalidate the use of the phantom as a reference standard for the purpose of this study. It would obviously not invalidate the in vivo correction which is obtained simultaneously with the clinical flow measurement. Therefore we excluded from further analysis those studies in which the velocity offset in the phantom did not agree with the velocity offset as observed in the stationary thorax wall in the in-vivo acquisitions within 0.6 cm/s (the phantom measurement accuracy check described in detail later in Methods).

### Image analysis

Initial analysis was carried out at each participating site, where analysis of the cardiac output without offset correction was performed. An ROI at the vessel of interest was manually set at each cardiac phase, using the magnitude image. By copying these ROIs to the velocity images, the time-integrated volume flow was assessed. This analysis was performed with the regular flow analysis packages in routine use at each site.

Subsequent analysis was performed at the core-lab (MJAR, MBMH at VUMC, NL) as follows.

### Interpolation-based offset correction method

**Fig. 1:**
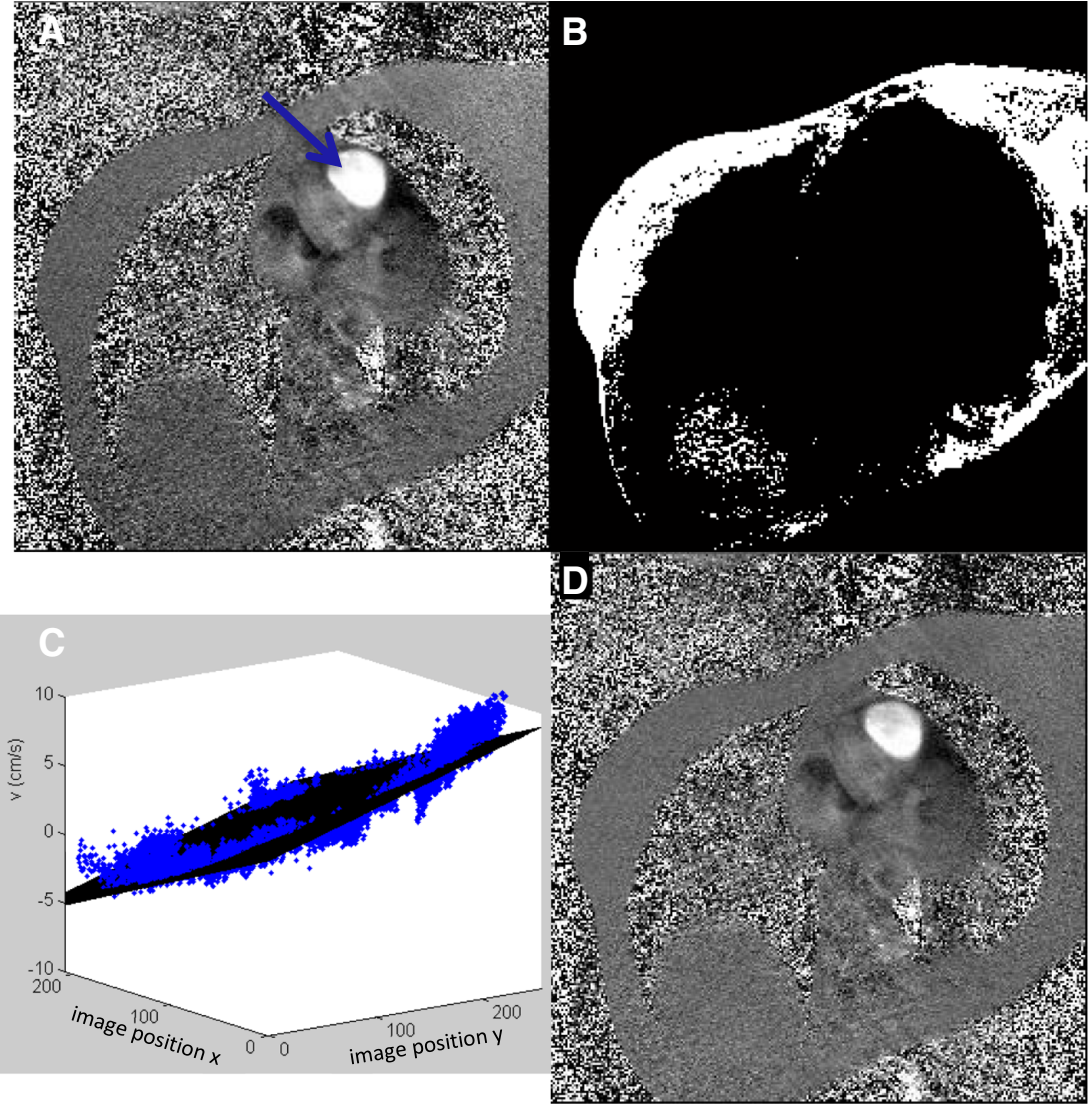
The velocity offset correction shown in a case of pulmonary artery (PA) flow assessment: original velocity map with the PA in cross-section (arrow) (**a**), mask of stationary pixels (**b**), 3D graph with fitted first order plane (in black) to the velocity values in the mask (blue points) showing the variation in background offset over the field of view (**c**) (see text) and corrected velocity map (**d**). Visually the corrected map shows that the left to right gradient in offset has been removed. (These images are shown with a small zoom which is why no PE FOV wraparound is seen)

For all in-vivo measurements an interpolation-based offset corrected dataset was obtained. The interpolation-based offset correction method was the method as described by Walker et al. [8], and earlier implemented for a single center study [14]. First, a pixel mask of stationary tissue was obtained by taking the 15% of pixels which had the lowest temporal variance of their velocity over the cardiac cycle (Fig. 1b). This stationarity percentile of 15% was set equal to original setting of Walker et al. [8]. A linear (first order, tilted but not curved) surface fit of the time-averaged velocity values within this mask was obtained as estimation of the velocity offset field (Fig. 1c). All of the velocity maps were corrected by subtraction of the fitted velocity offset field from the original velocity image, creating an offset corrected image series (Fig. 1d). This algorithm was implemented in Matlab (The Mathworks Inc., Natick, Massachusetts, USA).

In support of the second stated objective of this paper, the surface fitting was also performed using not only tilted planar surfaces (“linear fitting”) but also allowing tilted and curved surfaces (“higher order fitting” to 3rd order inclusive) (14).

In CMR acquisitions with phase-encode spatial wraparound, within the “overlapping” limit explained above, the areas of infolding tissue were manually traced and excluded from the image, before the 15% threshold on number of pixels was set to obtain the mask. To make the algorithm stable for images from all vendors, the algorithm was slightly adapted, because single pixels in the air and at the edges of the images appeared in the mask of stationary tissue using data from some vendors. First, to exclude zero or constant-filled pixels occurring in some reconstructions (e.g. filling in at FOV-edges after gradient distortion correction “image warping”), pixels with a temporal variance of the velocity over the cardiac cycle below 10^− 6^ cm/s were excluded before creating the mask. Second, to reduce sensitivity to noise pixels in air and tissue:air boundaries, the initial threshold was set above the 15%, and the resulting mask was eroded by 1 pixel, such that the final mask fit to the stationarity percentage of 15%.

**Fig. 2:**
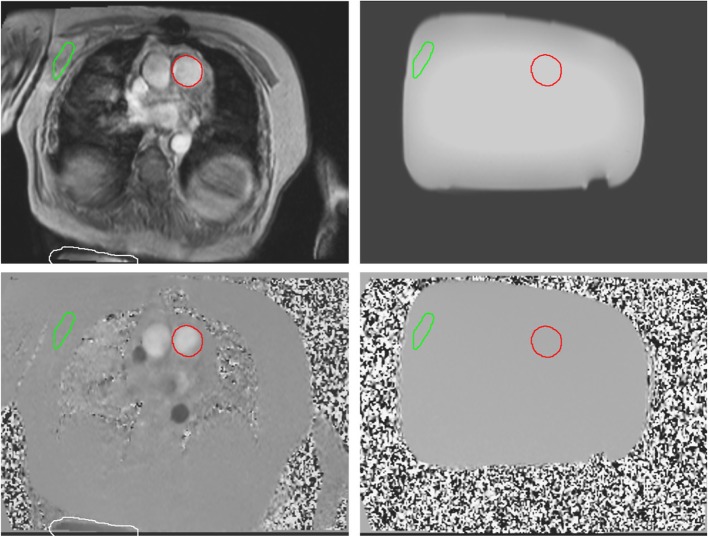
Example of in-vivo images (left) with corresponding phantom images (right), with magnitude images (top) and velocity images (bottom). The ROI’s at the vessel of interest (red), at the thorax wall (green) only for ‘the phantom measurement accuracy check’ (see Methods) and at an area of spatial wraparound (white) are shown. The white ROI was only set in a subset of the data when limited spatial wrap around was present

Please note that the variation of offset across the field of view is not supported by some commercial correction software, where a single static tissue ROI is placed and used for correction on the strongly inadequate assumption that the background correction is a constant across the field of view (i.e. the plane in C would not be tilted at all). That questionable method should not be confused with the single ROI used for the phantom measurement accuracy check explained later in the Methods and shown in Fig. 2.

### Assessment of velocity offsets

For each flow acquisition, an ROI was manually drawn at the vessel of interest at the first cardiac phase after the R-wave, at end-diastole (Fig. 2). The velocity offset estimated by the interpolation-based offset correction method at the position of the vessel (V_IB offset correction ROI vessel_) was assessed by copying the end-diastolic vessel ROI on the fitted velocity offset field of the offset correction. Subsequently, this ROI was copied to the phantom images, averaging over all simulated cardiac phases, to obtain the phantom estimation of the velocity offset for this flow acquisition (V_phantom ROI vessel_). Using the velocity offset value from the phantom measurement, the initial velocity offset error (V_offset pre correction_) was determined, as well as the residual offset error after interpolation-based correction (V_offset after IB correction_) at the location of the aorta or main pulmonary artery, see Eqs. 1 and 2.

1 $$ {\mathrm{V}}_{\mathrm{offsetprecorrection}}={\mathrm{V}}_{\mathrm{phantomROIvessel}} $$

2 $$ {\mathrm{V}}_{\mathrm{offset}\ \mathrm{after}\ \mathrm{IB}\ \mathrm{correction}}={\mathrm{V}}_{\mathrm{IB}\ \mathrm{offset}\ \mathrm{correction}\ \mathrm{ROI}\ \mathrm{vessel}}\hbox{-} {\mathrm{V}}_{\mathrm{phantom}\ \mathrm{ROI}\ \mathrm{vessel}} $$

The velocity sign of the velocity offsets was set such that a positive offset resulted in an overestimation of the cardiac output. The interpolated-based correction was repeated with 2nd and 3rd order surface fits, besides the original linear fit, to test the correction performances of these different spatial orders of interpolation. Using the assessed velocity offsets and the site’s initial cardiac output measurement, the error in the clinically assessed cardiac output was calculated. For the cardiac output analysis, the lowest interpolation order with the required performance (i.e. < 0.6 cm/s difference from the phantom estimate) was chosen, because higher interpolation orders make the correction more sensitive to artifacts and noise.

Finally, we assessed the pulmonic flow/systemic flow (Qp/Qs) ratio both with and without interpolation-based correction in a subset of subjects (as this was not the initial setup of this study), where both aorta and main pulmonary artery data were obtained and which were not suspected clinically of having shunts.

#### Phantom measurement accuracy check

To verify the accuracy of the phantom offset determination, a check was performed in order to make the phantom data a valid reference. From previous studies it was known, that offset assessment by a separate phantom measurement is not a good standard in all cases due to lack of temporal stability [10, 13]. We added additional pre-measurements in the phantom imaging protocol to create a similar system temperature. Nevertheless, a final check in the data was performed to exclude irregular phantom offset data. Therefore, a ROI was manually placed within stationary tissue at the anterior thorax wall. The location was chosen avoiding phase-encode ghosting flow artifacts arising from the heart and great vessels (Fig. 2). We copied this ROI to the phantom image, ensuring that the ROI was completely within the phantom. In this ROI both the velocity offset in the in-vivo acquisition as well as the phantom acquisition were determined, taking the averaged values over all cardiac phases (and over the ECG-simulated cardiac phases in the stationary phantom). Only studies with an agreement within 0.6 cm/s between these two values passed the phantom measurement accuracy check and were included in the Results.

### Statistical analysis

From the Methods section, it should be recalled that the velocity offset is an average over the entire cardiac cycle (since this is retro-gated cine imaging). Two average values of the velocity offset for each of the 6 systems were calculated, pre-correction and post-correction, by averaging over the studies collected on the system. These values are reported as mean ± standard deviation. The error in the cardiac output due to the velocity offset is expressed as a relative error in %. Differences in mean values in offset correction between the phantom and interpolation-based correction were tested with a paired Student’s t-test. Offsets were reported per system both as mean as well as using root mean square (RMS) values. RMS values show a better indication of the offset in an individual subject, whereas the normal average values indicate systematic offsets in a group of subjects. Interpolation order was judged by calculating the root mean square error per system. Values were compared to a target limit of 0.6 cm/s. Differences in variance were tested with an F-test. A *p* value < 0.05 was considered significant.

## Results

A total of 132 studies were sent to the corelab. We excluded 6 studies; 4 showing too much spatial wraparound in the phase encoding direction, 1 with incorrect phantom positioning, and 1 with the vessel not clearly in view. The remaining 126 flow measurements in 82 subjects were included; 46 male, age 43 ± 20 years. Fifty-nine measurements were at the main pulmonary artery, and 67 at the aorta. At 5 systems mostly clinical patient studies were included, whereas in the remaining site patients were included which fitted in another research protocol. Within each image series, areas of spatial wraparound in the phase encoding direction were manually excluded in 34 flow acquisitions.

**Fig. 3:**
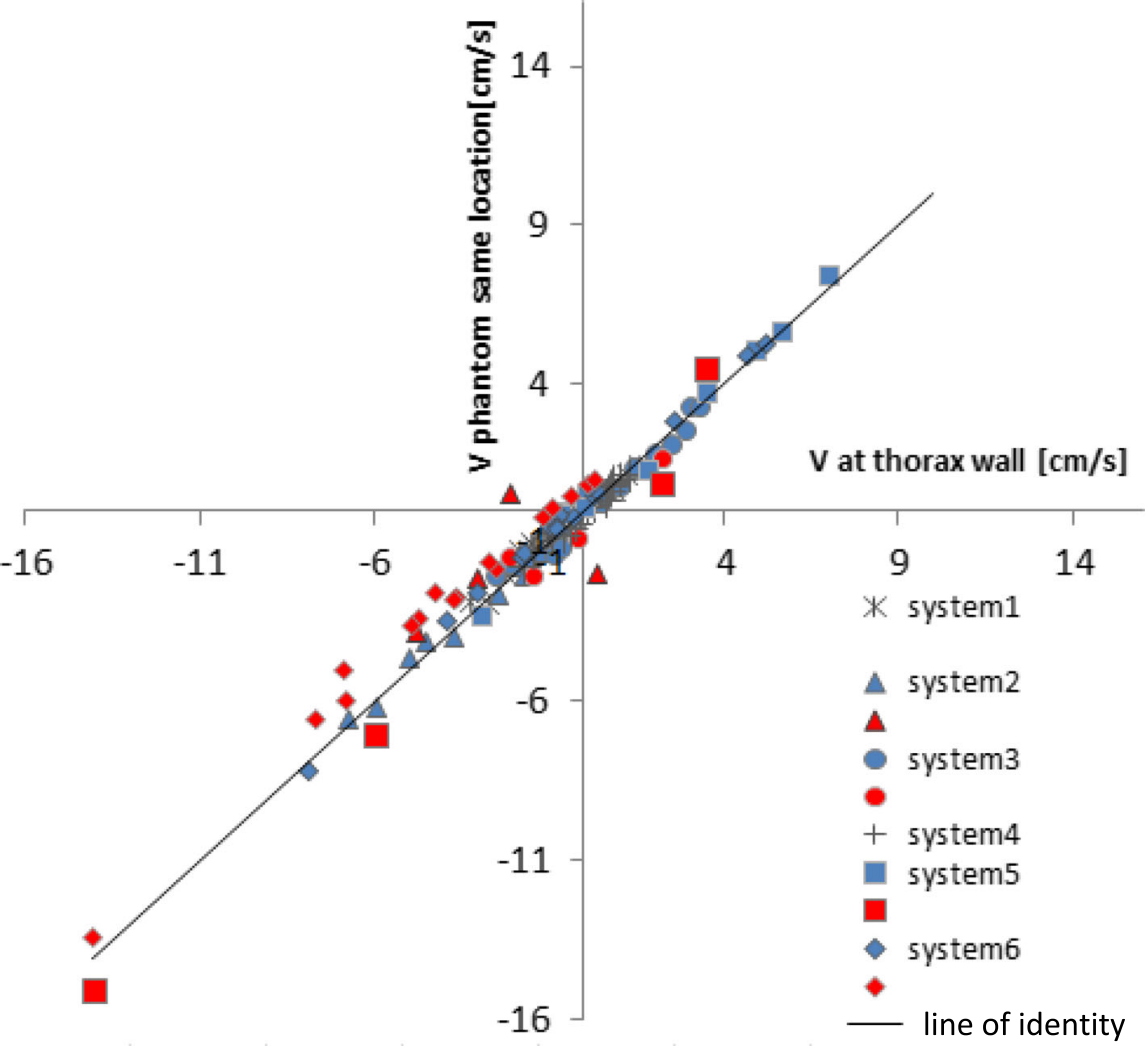
Velocity offset in a ROI of stationary tissue in the thorax wall compared to velocity as assessed in the phantom at the same location. Red symbols show the measurement points with a difference larger than 0.6 cm/s, which are excluded for further analysis; ‘the phantom measurement accuracy check’. For systems 1 and 4, no exclusions occurred. For other systems, the included points are shown in blue

For the ROI placed in stationary tissue in the thorax wall, there was overall (including all data) a good agreement between in-vivo and phantom scan of − 0.2 ± 0.6 cm/s (Fig. 3). However, as shown on Fig. 3, in total 28 studies were excluded due to a deviation larger than 0.6 cm/s between the phantom and in-vivo scan at the thorax wall, as defined in the Methods under phantom measurement accuracy check. Further analysis was performed with 98 studies. Table 2 shows the number of inclusions for the different systems.

**Table 2:** Number of patient studies per system

	# inclusions aorta	#inclusions MPA
System 1	10 (10)	10 (10)
System 2	9 (8)	9 (6)
System 3	10 (9)	10 (7)
System 4	10 (10)	10 (10)
System 5	10 (8)	5 (3)
System 6	18 (8)	15 (9)
Total	67 (53)	59 (45)

In brackets the number of inclusions with an agreement at the thorax wall with the phantom measurement < 0.6 cm/s. MPA, main pulmonary artery

**Fig. 4:**
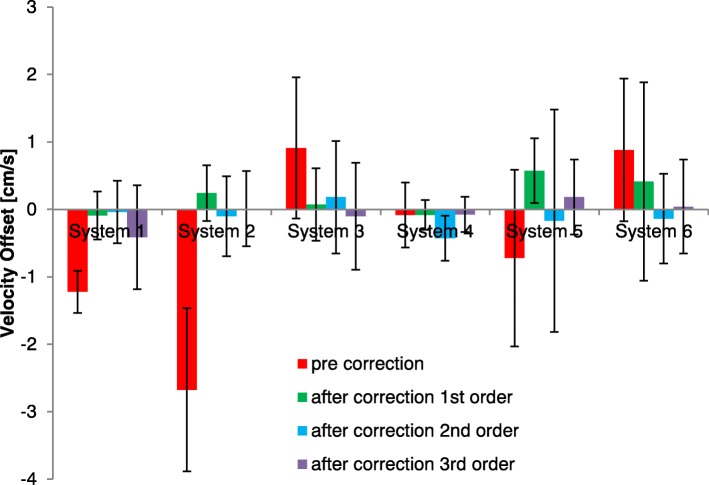
Velocity offset at aorta and main pulmonary artery before (V_offset pre correction_) and after offset interpolation-based correction (V_offset after IB correction_) with different orders of interpolation (mean and SD per MR system)

Before correction, the offset at the vessel (V_offset pre correction_) was − 0.4 ± 1.5 cm/s, and resulted in a − 5 ± 16% error in cardiac output. 40% of the patient studies showed a V_offset pre correction_ at the vessel smaller than ±0.6 cm/s, with a range of − 4.2 to 3.9 cm/s. Figures 4 and 6 show the offset at the vessel location and the resulting error in cardiac output for the different systems.

**Fig. 5:**
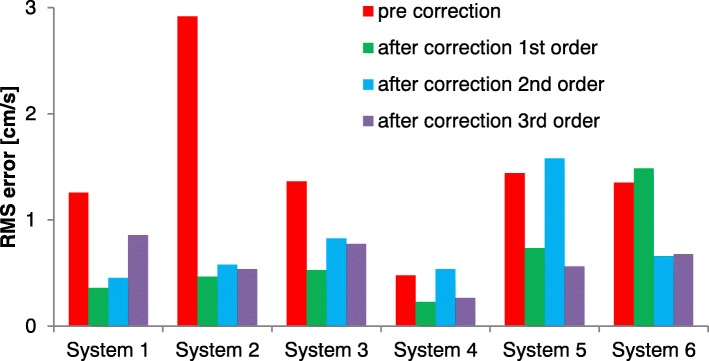
Root mean square (RMS) error of velocity offset (all aorta and main pulmonary artery results) per CMR system before (V_offset pre correction_) and after interpolation-based offset correction (V_offset after IB correction_) with different spatial orders of interpolation

**Fig. 6:**
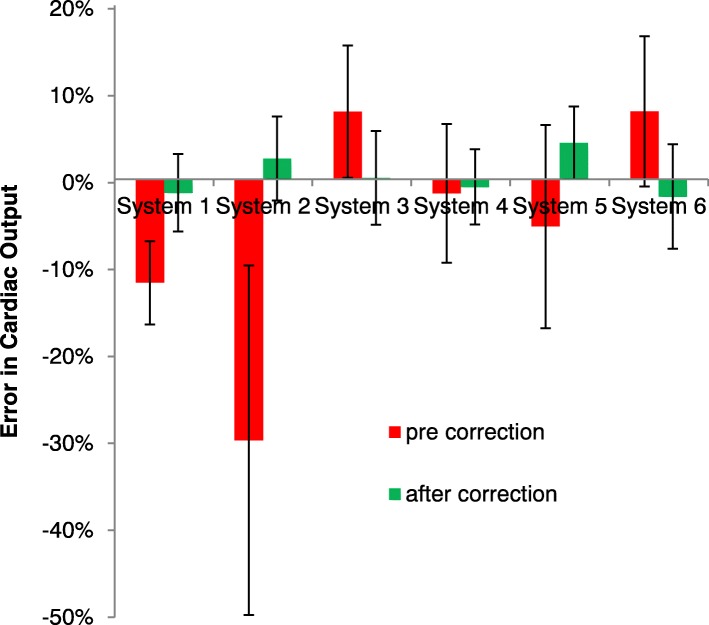
Relative error in cardiac output (all aorta and main pulmonary artery results) before and after interpolation-based offset correction (mean and SD per CMR system), with first order interpolation, except for system 6 with 2nd order interpolation

With interpolation-based correction the offsets (V_offset after IB correction_) decreased, as can be observed in Fig. 4 which shows the resulting offsets after correction using different spatial orders of interpolation. However, this figure is less clear for judging the optimal spatial order of interpolation, as both the mean and deviation are changing. In Fig. 5 the RMS error is plotted for the different spatial orders of interpolation. Here, it shows that first order interpolation clearly decreases the RMS error except for one GE system, system 6. Therefore for the further analysis of the overall effectiveness of correction, a first order correction was chosen for systems 1–5 and a 2nd order correction for system 6. Separate analyses for each vessel (main pulmonary artery/aorta) per system gave similar results for Figs. 4 and 5. The resulting errors in cardiac output are presented in Fig. 6.

After application of the interpolation-based correction with spatial orders as described above for each system, the remaining offset velocity (V_offset after IB correction_) was 0.1 ± 0.5 cm/s (*p* = 0.01) and 0 ± 5% error in cardiac output (*p* = 0.01), significantly smaller than before offset correction. Also the variance in offset over the subjects was significantly less after correction (*p* < 0.001). 78% of the individual measurements showed remaining error below 0.6 cm/s, with remaining errors were in the range of − 1.2 to 1.4 cm/s post correction. There was no statistical difference between the velocity offset assessed by the phantom or interpolation-based correction (*p* = 0.3).

The Qp/Qs was determined in a subset of 22 subjects which were not suspected clinically of having shunts and with measurement data in both vessels. The Qp/Qs was 1.1 ± 0.2, 1.03 ± 0.12 (*p* = 0.5), 1.02 ± 0.11 (*p* = 0.3) for the uncorrected, phantom-based correction, and interpolation-based correction, respectively (with *p* values tested with a paired t-test against the uncorrected Qp/Qs values).

## Discussion

In this study we have shown that without velocity offset correction, significant errors in cardiac output can occur in 2D phase contrast velocity quantification in the aorta and pulmonary artery, as reported before [1, 3, 4] and which occurred in 60% of the included scans in this study. The multi-vendor, multi-center setup allowed a broader evaluation of performance of the cited method. We validated the interpolation-based offset correction to be accurate within 0.1 ± 0.5 cm/s, as assessed over different centers and vendors. The interpolation-based offset correction is at least as accurate as the phantom based offset correction. The resulting error in cardiac output is small enough to support reliable use of this technique in clinical practice.

### Qp/Qs ratio

Earlier studies used the more clinically oriented Qp/Qs ratio, because this value is expected to be around 1.05 in patients not suspected of shunts [3, 6, 19]. While Qp/Qs was not the initial setup of this study, we further assessed its value in an subset. We did not detect a significant change in Qp/Qs with correction, probably due to low numbers, but the variance over the subjects was significantly decreased after interpolation-based correction (*p* = 0.01). Rigsby et al. also found inconclusive results for change in the Qp/Qs ratio in a single-center study with a similar interpolation-based offset correction [19], but found improvement by correction in comparing the main pulmonary artery flow with the combined flow in the left and right pulmonary arteries [19]. This might be explained by the system and protocol dependence of velocity offset errors, in that using a specific protocol on a specific system offsets are sometimes small, as can be seen in our results for system 4. Also Meierhofen et al. used the Qp/Qs ratio in 24 subjects in a single center study and concluded that, according to a normal ratio range of 0.9–1.2 that more patients without shunts incorrectly showed a calculated shunt after phantom-based correction [12]. Applying that same normal range in our study, we saw that interpolation-based correction removed incorrect shunts in 6 cases, and created in 2 cases a shunt (which we have not checked), and therefore improved the overall results.

### Spatial order of interpolation

In this study we examined the order of spatial interpolation of the offset fit to the mask pixels to the rest of the image including pertinently the location of the vessel of interest. The initial implementation of Walker et al. [8] applied a linear interpolation. Others have claimed later that higher spatial orders of interpolation should be applied [9, 15, 16]. Lankhaar et al. tested higher interpolation order for the pulmonary artery in a single center study, and found that errors increased for higher order fits [14]. In this study we also found that for 5 out of 6 CMR systems the RMS velocity error across all scans increased for 2nd order fitting compared to linear fitting. It is not that the velocity offset field per se is completely linear for systems 1–5, for in several patient studies these systems also showed offset fields with components of 2nd and 3rd order spatial variations. However, by making the interpolation-based correction operate at higher order instead of linear, the interpolation method became more sensitive to any other variation of the velocity error across the image FOV, resulting in greater variability over the group of studies as a whole. Among other factors, for higher-order fitting the error sensitivity increases to noise in the image, errors in the stationary mask, and small amounts of missed spatial wrap around. Giese et al. observed in phantom measurements that the largest component was linear and that 2nd order correction contributed much less [9]. Only for system 6 in our results (GE), linear interpolation increased the error and the clearly more effective 2nd order interpolation was applied in the final cardiac output results. A similar GE system 5 in our results showed also a slightly smaller RMS error by 3rd order interpolation, but we did not apply this to the cardiac output assessment because 3rd order interpolation makes the technique too sensitive to other errors in this setting. An earlier study on a GE system also included higher order terms in its correction algorithm [16]. A possible explanation may be imperfections in the Maxwell term correction, as those show primarily 2nd order spatial variations. Thus the interpolation-based offset correction method would require finally some tuning depending on the CMR system it is applied to, but this is probably a system/software specific property that can be more generally assessed.

Finally, these conclusions are for the aorta and main pulmonary artery, which usually are situated reasonably near the center of the FOV, and for every measurement the patient table was adjusted to place the FOV center in the z = 0 plane. To reduce offsets it would be better to put the vessel of interest at the z = 0 plane. However, this would require user input and manufacturers did not implement this. Placing the vessel of interest at the FOV center is not desirable, as this will induce spatial wraparound or requires a large FOV. Placing the FOV center in the z = 0 plane can be easily implemented in the regular workflow. For vessels further away from the center higher order corrections might be required. This is also the situation for velocity quantification in 4D Flow [20]; velocity quantification in this application includes vessels further away from the isocenter. The 3D slab of images includes more stationary tissue and spatial information so this application may benefit from higher order spatial interpolation depending on the specific system [5, 20, 21], but is outside the scope of the present study. Busch et al. published a recent study on this [17].

### Setting the mask of stationary pixels

Accurate and reliable determination of the mask of stationary pixels is essential to the interpolation-based correction. Here, we set the stationarity percentile to 15% as in the original paper of Walker et al. [8]. In the study by Lankhaar et al. [14] this stationarity percentile was set to 25%, but the dependency of offset error on stationarity percentile was shown to be minor in that study. We visually checked all masks to ensure that stationary tissue in the mask appeared on at least two sides of the thorax. This was the case in all 98 included studies. Secondly, we checked the mask visually for the presence of vessel or heart structures, as the genuine velocity data in these would corrupt the fitting which should only be to stationary tissue. We found the mask to be too large in 6 cases at 3 systems, partly due to the use of a relatively large FOV, which caused a large number of image pixels completely outside the subject, so in these cases a stationarity percentile of 8–10% was more reliable. However, 15% was consistently used for the main results presented as the presence of some heart or vessel pixels in the mask in these cases did not have any noticeable influence on the results.

### Implementation of the interpolation-based correction

The algorithm as applied in this study can be implemented in an analysis package for CMR flow post-processing, as is currently already the case for some commercial packages. In principle, the algorithm can run without strict supervision. Manual supervision is still required for the exclusion of phase-encode spatial wrap around before generating the pixel mask. Secondly, the user would also be required to confirm that stationary tissue is included in the mask to both sides of the thorax, and that moving structures of the heart and large vessels (and occasionally great veins with slow non-pulsatile flows) are excluded. Metallic implants are also an increasingly common problem in clinical work with some predictable difficulty in handling nearby pixels. The analysis package could display the stationary pixel mask, so the user could adjust the stationarity percentile as described above. Finally, it may be necessary as in this study that the spatial order of interpolation has to be set per CMR system.

### Limitations

A limitation of the interpolation-based offset correction is that it is sensitive to phase-encode FOV wraparound (spatial aliasing). Ideally this should be prevented in scanning, but this is quite a stringent limitation and tends to increase breath-hold time, unless other sequence parameters are adjusted, with all their consequences. If phase-encode wraparound does not reach as far as overlapping the direct image, or overlaps only small regions, it can be excluded manually before correction. However, any such dependence on intervention can be an obstacle to reliable clinical use. For some protocols it can be helpful to increase the phase-encoding FOV using parallel imaging [22], because it can be difficult to sufficiently control phase-encode aliasing, especially for the typical oblique angulation of the cross-section of the pulmonary artery.

A second protocol optimization specific for this interpolation-based correction is to ensure that the posterior RF receiver coils are switched on, even in cases where the vessel of interest, such as the main pulmonary artery is in the anterior part of the thorax. The posterior coils are not of interest for the SNR at the level of the vessel, but they are essential to provide sufficient SNR for the CMR signal of the stationary tissue on the posterior side of the thorax.

In this study we considered only 1.5 T whole body superconducting CMR systems. Most CMR studies are still performed at this type of CMR system. However, there is an increased application of 3 T for CMR. The origin of the velocity offset is within the gradient system and its associated errors, and the overall specification of these does not change at 3 T (because it is already close to the limit of nerve stimulation), except that Maxwell terms are smaller, so any imperfections in their software correction might be expected to reduce as well. Therefore, we expect that these results should also be valid at 3 T CMR systems. However, another aspect in which these systems might deviate physically from 1.5 T is mechanical vibration, as the mechanical force for the same gradient performance scales with main field strength. A recent small single-center study at 3 T found similar results as this study considering Qp/Qs ratios [23].

Care should be taken not to interpret the specific offsets found in this study as definitely linked to particular vendor models of CMR systems. In this study we used on purpose as much as possible each site’s customary CMR protocols, thus specific settings such as breath-holding, typical slice orientation and measurement location at the vessel of interest would be likely to give a strong bias on the velocity offset observed using the different CMR systems [11]. The absence of any rigorous set of scanning parameters means that this study cannot be applied to compare velocity offsets between CMR systems and such comparison was not part of the study design.

Notwithstanding the above, we noticed incidentally that the two Philips CMR systems (1,2) in this study showed a relatively high uncorrected velocity offset. Besides the issue of the protocol differences between sites as mentioned before, one should realize that some systems may apply in their default protocols a background phase-offset correction filter. On Philips machines this is known as the ‘LPC filter’, developed to reduce the phase offset in CMR contrast angiography, where it is expected to reduce the velocity offset [24]. On the other hand, there is no published validation study for the application of this filter for vessels around the heart. The applied filter kernel is expected to be influenced to some unknown extent by the presence of sufficient non-stationary tissues around the vessel of interest. Due to the principles of the interpolation-based offset correction and the ‘LPC filter’, a combination of applying the LPC filter before the interpolation-based correction is not useful to test. Therefore, we switched the ‘LPC filter’ off on Philips systems.

Retrospective ECG gating was used in this study. In case of unstable heart rhythms, prospective ECG gating shows clinically more reliable measurements. In the case of prospective ECG gating it has been shown that the amount of velocity offset varies with the timing after the sequence starts running in each cardiac cycle, and tends to be larger directly after the ECG trigger [9]. The interpolation-based offset correction should then be implemented per cardiac phase, instead of the time-averaged offset value as in this study. Giese et al., implemented this using linear correction, but did not report good results [9]. This might need additional validation as the variation per phase after sequence start is highly complex dependent on incomplete sequence spoiling [25]. However, this is solved on newer systems as prospective ECG gating keeps on running the sequence continuously while watching the ECG. At some centres it was not possible to set a simulated ECG to the patient heart-rate and sometimes 60 bpm was used. Because of the use of retrospective cine gating, we do not expect the difference in heart rate to cause any change in the velocity offset, between the in-vivo measurement and the phantom measurement.

In this study, the velocity offset was assessed at the vessel position in the first cardiac phase. In reality the vessel position varies somewhat over the cardiac phases. This effect was neglected, but the phantom data show that the spatial variation of the offset in this motion range is limited.

The ‘phantom measurement accuracy check’ was performed with a relatively small ROI. Ideally, we would perform this check on the total mask of stationary tissue. However, this would require a stationary phantom of the size of a large adult thorax at all centers. Because of practical reasons, we were compelled to use a smaller phantom allowing only the vessel and a part of the anterior thorax wall to be covered by the phantom. Instead of the phantom, the possibility of some form of “internal validation”, for example using LV stroke volume from cine stack, or by Qp/Qs, was limited by requiring definitely normal subjects, by other well-known sources of inaccuracy and also by having no permission to make any extra patient acquisitions for this unfunded work.

The study protocol was open on the use of breath-hold or non-breath-hold techniques, as well as the precise acquisition parameters and method of positioning the measurement plane in the two vessels. Every site applied the technique in their own regular manner (this was necessary for recruitment at all without funding). Therefore, this study shows a realistic variability of protocol settings, but of course this might have introduced also variations between the different sites and systems. We emphasise that the acquisition parameters were checked as identical for each in-vivo scan and its phantom scan, so controllable differences there were not a source.

Even in nominally breath-hold scans, phase-encode ghosting artefacts of the bright superficial tissues (especially fat) are often problematic, and furthermore might sometimes be relatively constant over the cardiac cycle (depending on a few factors not to go into here), and so the impact of artefacts on chest wall phase might get past the temporal variance test of the interpolation-based correction method (which aims to exclude flow ghosting as described in Methods). It is uncertain whether this corrupting effect is usually small (because the subtracted reference and velocity-encoded scans are normally almost simultaneous in terms of the respiratory motion) compared to the true pixel brightness at the wall, and is of some possible concern (as are other variations [25]) because the background offset we seek to correct is also often small.

Finally, the subset of Qp/Qs studies was acquired at many different centres where the placement of the aortic plane might have varied relative to the coronary ostia, that can require a few % correction in the ‘normal’ Qp/Qs value.

### Future

In future CMR systems, field camera measurements of real-time effective gradient fields might enable better compensation of phase offsets [9, 26], but this potential advance faces significant challenges including the distribution of field camera probes external to the thorax and the insufficient SNR without averaging for enough accuracy to support typical background phase errors. Using non real-time separate calibration measurement for this compensation would only work when the temporal stability of the systems is good enough, so it could be included in regular CMR maintenance measurements.

## Conclusions

This study shows that interpolation-based velocity offset correction reduces the offset with comparable efficacy as phantom-based offset correction, without the time penalty imposed by separate phantom scans with their associated concern regarding short-term thermal stability. This method showed to be stable for 2D retrospective ECG triggered phase-contrast velocity quantification in the large vessels around the heart. However, some manual intervention in the largely automated correction of patient scans remained necessary, and the optimum spatial order of interpolation required initial assessment for each type of system, because linear was not always optimal.

## Abbreviations

2D: Two dimensional
CMR: Cardiovascular magnetic resonance
ECG: Electrocardiogram
FOV: Field-of-view
LPC: Low pass correction
Qp: Pulmonic flow
Qs: Systemic flow
RF: Radiofrequency
RMS: Root mean square
ROI: Region-of-interest
SNR: signal to noise ratio
V_enc_: Encoding velocity
V_offset_: Offset velocity
V_peak_: Peak velocity
